# Persistent Pulmonary Hypertension of the Newborn: The Efficacy Comparison of Vasodilators Sildenafil Plus Bosentan Versus Sildenafil Plus Beraprost at a Tertiary Childcare Health Facility

**DOI:** 10.7759/cureus.20020

**Published:** 2021-11-29

**Authors:** Mudasser Adnan, Muhammad Sohail Arshad, Hafiz Muhammad Anwar-ul-Haq, Munir Ahmad, Hira Afsheen, Hashim Raza

**Affiliations:** 1 Department of Pediatric Cardiology, The Children’s Hospital & The Institute of Child Health, Multan, PAK; 2 Department of Neonatology, The Children’s Hospital & The Institute of Child Health, Multan, PAK; 3 Department of Pediatric Nephrology, The Children’s Hospital & The Institute of Child Health, Multan, PAK

**Keywords:** persistent pulmonary hypertension of the newborn, echocardiography, beraprost, bosentan, sildenafil

## Abstract

Background

Persistent pulmonary hypertension of the newborn (PPHN) has been known for more than three decades, and lots of advancements have been made regarding its diagnosis and management. However, the exact causes of PPHN and the best treatment strategies remain debatable. This study aimed to compare the effectiveness of sildenafil and bosentan versus sildenafil and beraprost in the management of persistent pulmonary hypertension of the newborn (PPHN).

Methodology

This open-label, non-randomized, quasi-experimental study was conducted at the Department of Pediatric Cardiology and Neonatology, The Children’s Hospital & The Institute of Child Health, Multan, Pakistan, from January 2021 to June 2021. We enrolled a total of 50 newborns (25 in each group) aged <10 days, gestational age above 34 weeks, who presented with respiratory distress and marked hypoxemia (PaO_2_ < 50 mmHg) as per arterial blood gas (ABG) analysis and confirmed echocardiographic diagnosis of PPHN within 24 hours of admission. A total of 25 cases were given sildenafil and bosentan, while the other 25 cases were given sildenafil and beraprost. Echocardiographic examination was done again after 72 and 120 hours, and the findings were noted. Outcomes were measured in terms of the reduction in tricuspid regurgitation (TR), mortality, and duration of hospital stay.

Results

Of the 50 neonates, 27 (54.0%) were male. Overall, the mean age was calculated to be 3.54 ± 0.7 days. The mean gestational age was 35.0 ± 0.7 weeks. The mode of delivery was cesarean section among 35 (70.0%) neonates. A significantly higher reduction in tricuspid regurgitation after 72 and 120 hours following the initiation of the treatment was observed in the sildenafil plus bosentan group in comparison with the sildenafil plus beraprost group (p < 0.05). No statistically significant difference was observed in terms of the duration of hospitalization between both study groups (p = 0.1776).

Conclusion

The combination of sildenafil and bosentan was found to be more effective than sildenafil and beraprost in reducing tricuspid regurgitation after 72 hours, while they have comparable efficacy at 120 hours of treatment in the management of persistent pulmonary hypertension of the newborn.

## Introduction

Persistent pulmonary hypertension of the newborn (PPHN) is described as an increase in the pulmonary vascular resistance and right-to-left shunt at the atrial and ductal level accompanying severe hypoxemia [1]. PPHN is a complicated and life-threatening condition and is considered to be a difficult-to-treat disease. The incidence of PPHN is estimated to be between 2 and 6.8 per 1,000 live births, while mortality ranges between 10% and 20% [2].

PPHN has been known for more than three decades, and lots of advancements have been made regarding its diagnosis and management. However, the exact causes of PPHN and the best treatment strategies remain debatable. The use of newly available drugs for the treatment of PPHN is based on experimental endorsements or findings of results in adult age groups suffering from persistent pulmonary hypertension. Generally, an aggressive approach for supporting cardiac functioning and perfusion along with volume and inotropic drugs to improve cardiac output and systemic O_2_ transportation is essential in PPHN, while the main principle of treating PPHN is selective pulmonary vasodilation [3,4]. Pulmonary vasodilators such as magnesium sulfate, sildenafil, bosentan, prostacyclin, and beraprost are some of the most commonly adopted options for the treatment of PPHN [5]. Inhaled nitric oxide (iNO) and extracorporeal membrane oxygenation (ECMO) are considered to be the “gold standard” for the treatment of PPHN, but as these are expensive options and linked with technical expertise in resource-limited settings, there is always a need for relying on affordable therapeutic options that assure quick efficacy and stabilization for cases of PPHN [6]. As no single treatment option is described as a magic drug in PPHN, there lies a huge requirement for analyzing the effectiveness and safety of the currently available treatment options. A local study published in 2018 comparing the effectiveness of sildenafil alone versus sildenafil and bosentan in PPHN revealed that tricuspid regurgitation (TR) was significantly lower among newborns using sildenafil plus bosentan (41.66 ± 9.47 mmHg versus 53.30 ± 9.35 mmHg, p < 0.0001) [7]. A retrospective study from Thailand analyzing beraprost in PPHN stated that it can be considered as an alternative treatment option in PPHN [8]. To the best of our knowledge, no study has been conducted so far comparing the effectiveness of sildenafil plus bosentan versus sildenafil plus beraprost in PPHN; therefore, this study was planned.

## Materials and methods

Study design

This is an open-label, non-randomized, quasi-experimental study.

Place and duration of the study

This study was conducted at the Department of Pediatric Cardiology and Neonatology, The Children’s Hospital & The Institute of Child Health, Multan, Pakistan, from January 2021 to June 2021.

Inclusion criteria

Newborns aged <10 days, gestational age above 34 weeks, who presented with respiratory distress and marked hypoxemia (PaO_2_ < 50 mmHg) as per arterial blood gas (ABG) analysis and confirmed echocardiographic diagnosis of PPHN within 24 hours of admission were enrolled.

Exclusion criteria

Newborns with congenital heart disease other than atrial septal defect or small ventricular septal defect were excluded. Newborns presenting with seizures, sepsis, and pneumothorax or with congenital anomalies were also not included. Newborns requiring ventilation were also excluded.

Data collection

Approval from the Institutional Ethical Committee of The Children’s Hospital & The Institute of Child Health was acquired (reference #: 407/20; date: 7-3-2020). Informed written consent was sought from the parents/guardians of all study participants. Considering 95% confidence interval (two-sided), power of 80%, sample size ratio of 1:1, and P1 as 41.66 ± 9.47 and P2 as 53.3 ± 9.35 [7], a sample size of 22 patients was calculated. We enrolled a total of 50 newborns (25 in each group) as per the inclusion and exclusion criteria. Confirmation of echocardiographic diagnosis of PPHN was based on right-to-left or bidirectional hemodynamic shunting at the ductus arteriosus or patent foramen ovale (PFO) and TR jet pressure above 40 mmHg. All echocardiography examinations were performed by a pediatric cardiologist with a post-fellowship experience of more than three years.

At the time of admission, the gender, age (days), birth weight (grams), gestational age (weeks), mode of delivery, APGAR score at five minutes, and echocardiographic findings of all newborns were recorded. As per the inclusion and exclusion criteria, in the 50 newborns, the first 25 consecutive cases of PPHN were allocated to group A (sildenafil plus bosentan), while the remaining 25 cases were allocated to group B (sildenafil plus beraprost). In group A, the newborns were given sildenafil 1 mg/kg/dose thrice a day along with bosentan 1 mg/kg/dose twice a day. In group B, sildenafil was given 1 mg/kg/dose thrice a day along with beraprost 1 ug/kg/dose thrice a day. Echocardiographic examination was done again after 72 hours and 120 hours, and the findings were noted. Outcomes were measured in terms of the reduction in tricuspid regurgitation (TR), mortality, and duration of hospital stay. The newborn’s discharge criteria were clinical stability and starting feeding orally. The side effects of the treatment were noted in both study groups. No sponsorship or funding was acquired for this study, and there was no conflict of interest between the researchers of this study.

Data analysis

A customized proforma was designed to record the study data, whereas SPSS version 26.0 was used for data analysis. Age (days), birth weight (grams), gestational age (weeks), APGAR score at five minutes, tricuspid regurgitation (mmHg) at baseline and after 72 and 120 hours, and the duration of hospitalization were represented as mean and standard deviation (SD). Gender and the mode of delivery were represented as frequency and percentage. Between study groups, quantitative data were compared using independent samples Student’s t-test, while qualitative variables were compared using the chi-squared test. A P-value of less than or equal to 0.05 was considered significant.

## Results

Of the 50 neonates, 27 (54.0%) were male. Overall, the mean age was calculated to be 3.54 ± 0.7 days. The mean gestational age was 35.0 ± 0.7 weeks. The mode of delivery was cesarean section among 35 (70.0%) neonates. The mean birth weight was noted to be 2189 ± 164 g. Table 1 shows the comparison of baseline characteristics among the neonates of both study groups, and no statistically significant difference was noted (p > 0.05).

**Table 1:** Characteristics of the Newborns in Both Study Groups (n = 50)

Characteristics		Sildenafil plus bosentan (n = 25)	Sildenafil plus beraprost (n = 25)	P-value
Gender	Male	14 (56.0%)	13 (52.0%)	0.7766
	Female	11 (44.0%)	12 (48.0%)	
Age in days ( mean ± SD)		3.68 ± 1.50	3.41 ± 1.62	0.5438
Gestational age in weeks (mean ± SD)		35.0 ± 0.6	34.9 ± 0.8	0.6194
Mode of delivery	Cesarean section	18 (72.0%)	17 (68.0%)	0.7576
	Vaginal delivery	7 (28.0%)	8 (32.0%)	
APGAR score at five minutes (mean ± SD)		8.5 ± 0.4	8.4 ± 0.3	0.3223
Birth weight in grams (mean ± SD)		2184 ± 156	2199 ± 172	0.7481
Tricuspid regurgitation in mmHg at baseline (mean ± SD)		63.8 ± 10.2	64.9 ± 9.4	0.6935
Baseline pulmonic insufficiency (mean ± SD)		26.6 ± 7.4	24.2 ± 7.1	0.2154
Baseline ejection fraction in % (mean ± SD)		62.8 ± 6.9	62.2 ± 6.2	0.1841

Tricuspid regurgitation after 72 hours was noted to be significantly lower in the sildenafil plus bosentan group in comparison with the sildenafil plus beraprost group (42.7 ± 8.2 mmHg versus 50.4 ± 9.8 mmHg, p = 0.0041). A significantly higher reduction in tricuspid regurgitation after 72 hours following the initiation of the treatment was observed in the sildenafil plus bosentan group in comparison with the sildenafil plus beraprost group (21.1 ± 2.0 mmHg versus 14.5 ± 0.4 mmHg, p < 0.0001). The combination of sildenafil plus bosentan showed an earlier response at 72 hours, while both groups had comparable efficacy at 120 hours. Although the P-value was significant, the reduction was not significantly higher as both combinations have almost similar efficacy at 120 hours (26.3 mmHg versus 23.7 mmHg), with a deference of 2.6 mmHg, which is not clinically significant as proven by the measured tricuspid regurgitation (TR) at 120 hours. Overall, the mean duration of hospital stay was 8.9 ± 3.4 days, while no statistically significant difference was observed in terms of the duration of hospitalization between both study groups (8.1 ± 3.1 days in the sildenafil plus bosentan group versus 9.4 ± 3.6 days in the sildenafil plus beraprost group, p = 0.1776). No mortality was reported in any of the cases. Table 2 shows the echocardiographic outcome comparisons between both study groups.

**Table 2:** Comparison of the Echocardiographic Findings (Mean ± SD) in Terms of Tricuspid Regurgitation, Mean Pulmonic Insufficiency, and Mean Ejection Fraction in Both Study Groups (n = 50)

	Echocardiographic findings	Sildenafil plus bosentan (n = 25)	Sildenafil plus beraprost (n = 25)	P-value
Tricuspid regurgitation in mmHg	After 72 hours	42.7 ± 8.2	50.4 ± 9.8	0.0041
	Reduction from baseline after 72 hours	21.1 ± 2.0	14.5 ± 0.4	<0.0001
	After 120 hours	37.5 ± 6.4	41.2 ± 7.2	0.0608
	Reduction from baseline after 120 hours	26.3 ± 3.8	23.7 ± 2.2	0.0048
Pulmonic insufficiency in mmHg	After 72 hours	15.7 ± 3.8	16.4 ± 3.5	0.5014
	Reduction from baseline after 72 hours	10.9 ± 3.6	7.8 ± 3.6	0.0038
	After 120 hours	8.6 ± 2.3	8.9 ± 2.5	0.6608
	Reduction from baseline after 120 hours	16.0 ± 5.1	15.3 ± 4.6	0.6127
Ejection fraction in %	After 72 hours	63.2 ± 5.3	63.5 ± 5.2	0.8408
	Increase from baseline after 72 hours	1.8 ± 1.3	1.3 ± 1.0	0.1340
	After 120 hours	66.8 ± 4.8	66.4 ± 4.6	0.7648
	Increase from baseline after 120 hours	4.0 ± 2.1	4.2 ± 2.6	0.7661

## Discussion

In 1969, Gersony and coworkers were the first to document pulmonary hypertension [9]. A recent review described sildenafil alone or in combination with iNO to be the best option for managing PPHN. However, iNO or ECMO are not widely available in developing countries such as Pakistan, while newborns with PPHN are commonly managed with sildenafil, bosentan, and beraprost along with supportive care at our center [5]. Supportive care such as maintenance of optimal temperature, nutrition, avoiding stress, and gentle handling are some of the important basic principles in the management of PPHN [10,11].

In the present study, we found that the combination of sildenafil and bosentan was significantly more effective in PPHN in comparison with the combination of sildenafil and beraprost. A local study from the same study center analyzing the effectiveness of sildenafil alone and in combination with bosentan revealed that the combination of sildenafil and bosentan was more efficacious than sildenafil alone in PPHN [7]. Various trials have found sildenafil to result in a significantly better oxygenation index among newborns with PPHN, while sildenafil is also found to increase survival rates among newborns with PPHN [12-14].

On the other hand, a study done by Steinhorn et al. evaluating the efficacy of bosentan as adjunctive therapy for PPHN concluded no additional advantage of bosentan when used with iNO in term newborns [15]. Some researchers have found the combination of bosentan and sildenafil to be effective when used as adjunctive therapy to iNO [16]. A recent study from Iran comparing the efficacy of bosentan versus sildenafil among infants with pulmonary arterial hypertension has shown that bosentan has comparable efficacy to sildenafil in terms of reducing pulmonary artery pressure as well as improving cardiac output [17]. In the present research, although the duration of hospitalization was comparatively low in the sildenafil and bosentan group in comparison with the sildenafil and beraprost group (8.1 ± 3.1 days versus 9.4 ± 3.6 days), the difference was not statistically significant (p = 0.1776). Most of the experimental trials evaluating the role of newer treatment options such as tadalafil, macitentan, ambrisentan, riociguat, and selexipag regarding the management of persistent pulmonary hypertension are limited to adult patients and showed no significant beneficial effects [18].

There were a few limitations of this study. As this was a single-center study with small sample size, further studies are needed to further establish the role of combination therapies for the management of PPHN. We were only focused on evaluating the reduction in tricuspid regurgitation among the current set of patients for evaluating the effectiveness of both treatment combinations, but we could not evaluate their effects on the main pulmonary artery diameter, mean pulmonary artery pressure, or right ventricular end-diastolic diameter. We also noted relatively short outcomes among newborns with PPHN. Further trials comparing the efficacy of different therapeutic options and their long-term effects should also be conducted.

## Conclusions

The combination of sildenafil and bosentan was found to be more effective than sildenafil and beraprost in reducing tricuspid regurgitation after 72 hours, while they have comparable efficacy at 120 hours of treatment in the management of persistent pulmonary hypertension of the newborn.

## References

[1] Arshad MS, Adnan M, Anwar-Ul-Haq HM, Zulqarnain A (2021). Postnatal causes and severity of persistent pulmonary hypertension of newborn. Pak J Med Sci.

[2] Lakshminrusimha S, Keszler M (2015). Persistent pulmonary hypertension of the newborn. Neoreviews.

[3] Razzaq A, Iqbal Quddusi A, Nizami N (2013). Risk factors and mortality among newborns with persistent pulmonary hypertension. Pak J Med Sci.

[4] Nakwan N, Jain S, Kumar K (2020). An Asian multicenter retrospective study on persistent pulmonary hypertension of the newborn: incidence, etiology, diagnosis, treatment and outcome. J Matern Fetal Neonatal Med.

[5] Pedersen J, Hedegaard ER, Simonsen U, Krüger M, Infanger M, Grimm D (2018). Current and future treatments for persistent pulmonary hypertension in the newborn. Basic Clin Pharmacol Toxicol.

[6] Agrawala A, Agrawal R (2013). Persistent pulmonary hypertension of the newborn: recent advances in the management. Int J Clin Pediatr.

[7] Fatima N, Arshad S, Quddusi AI, Rehman A, Nadeem A, Iqbal I (2018). Comparison of the efficacy of sildenafil alone versus sildenafil plus bosentan in new-borns with persistent pulmonary hypertension. J Ayub Med Coll Abbottabad.

[8] Nakwan N, Nakwan N, Wannaro J (2011). Persistent pulmonary hypertension of the newborn successfully treated with beraprost sodium: a retrospective chart review. Neonatology.

[9] D'cunha C, Sankaran K (2001). Persistent fetal circulation. Paediatr Child Health.

[10] Konduri GG, Kim UO (2009). Advances in the diagnosis and management of persistent pulmonary hypertension of the newborn. Pediatr Clin North Am.

[11] Al Mamun MA (2019). Sildenafil in the management of pulmonary hypertension in newborn. DS (Child) H J.

[12] Hussain AS, Ali R, Ahmed S, Naz F, Haroon A (2017). Oral sildenafil use in neonates with persistent pulmonary hypertension of newborn. J Ayub Med Coll Abbottabad.

[13] Barst RJ, Beghetti M, Pulido T, Layton G, Konourina I, Zhang M, Ivy DD (2014). STARTS-2: long-term survival with oral sildenafil monotherapy in treatment-naive pediatric pulmonary arterial hypertension. Circulation.

[14] Kelly LE, Ohlsson A, Shah PS (2017). Sildenafil for pulmonary hypertension in neonates. Cochrane Database Syst Rev.

[15] Steinhorn RH, Fineman J, Kusic-Pajic A (2016). Bosentan as adjunctive therapy for persistent pulmonary hypertension of the newborn: results of the randomized multicenter placebo-controlled exploratory trial. J Pediatr.

[16] Goissen C, Ghyselen L, Tourneux P, Krim G, Storme L, Bou P, Maingourd Y (2008). Persistent pulmonary hypertension of the newborn with transposition of the great arteries: successful treatment with bosentan. Eur J Pediatr.

[17] Farhangdoust S, Mehralizadeh S, Bordbar A (2020). Comparison of the effects of bosentan and sildenafil in the treatment of persistent pulmonary arterial hypertension in infants. J Clin Neonatol.

[18] Lajoie AC, Bonnet S, Provencher S (2017). Combination therapy in pulmonary arterial hypertension: recent accomplishments and future challenges. Pulm Circ.

